# Supplementary material for: Being Active during the Lockdown: The Recovery Potential of Physical Activity for Well-Being
Source: Int J Environ Res Public Health. 2021 Feb 10;18(4):1707. doi: 10.3390/ijerph18041707 (PMC7916567; doi:10.3390/ijerph18041707)
Supplement: Supplementary file 1 [file ijerph-18-01707-s001.zip › Supplemental file/Informed consent.pdf]

Mrs, Mr, Good morning,

The Sport and Social Environment Laboratory (EA3742) at the University of Grenoble Alpes is conducting a scientific study to understand physical activity practices during lockdown related to the coronavirus epidemic.

If you consent to answer this questionnaire, we guarantee the protection of your answers in accordance with the general regulations for the protection of personal data (GRPD). The data will be anonymous and may not be exchanged commercially under any circumstances. They will be archived securely at the University of Grenoble Alpes for a period of 15 years from the last date of data collection. For more information on the protection of your data, you can contact the Director of the Sport and Social Environment Laboratory, aina.chalabaev@univ-grenoble-alpes.fr.

If you click on the " Next " button below, you freely declare that you agree to participate in this research, and accept that your information will be used anonymously and confidentially for research purposes and, ultimately, for publication in scientific journals.

Sincerely,
The SENS Lab Team
