# Supplementary material for: Being Active during the Lockdown: The Recovery Potential of Physical Activity for Well-Being
Source: Int J Environ Res Public Health. 2021 Feb 10;18(4):1707. doi: 10.3390/ijerph18041707 (PMC7916567; doi:10.3390/ijerph18041707)
Supplement: Supplementary file 1 [file ijerph-18-01707-s001.zip › Supplemental file/Full correlation matrix.pdf]

Table 1. Correlation matrix for main variables included in the study

	M	SD	Observed Range	1	2	3	4	5	6	7	8	9	10	11	12	13	14	15
1. Sex	-	-	-															
2. Age	34.06	14.18	17-91	-0.08														
3. Number of children	0.62	1.12	0-7	-0.08	0.03													
4. Housing with or without outside space	-	-	-	0.06	0.01	0.36****												
5. House sizing	104.65	62.32	9-477	-0.08	0.08	0.29****	0.13*											
6. Face-to-face work	-	-	-	0.00	0.07	-0.04	0.01	0.05										
7. Teleworking	-	-	-	0.02	0.03	-0.09	-0.08	0.01	-0.39****									
8. MVPA	400.52	325.02	0-2130	-0.09	-0.07	0.08	0.06	-0.07	-0.06	-0.02								
9. Usual level of PA	3.02	0.91	1-4	-0.18**	-0.29****	0.07	0.04	-0.10	-0.08	-0.03	0.34****							
10. Detachment	4.61	1.41	1-7	0.10	-0.08	0.07	0.08	0.00	-0.01	0.06	0.24****	0.08						
11. Relaxation	5.26	1.28	1-7	0.03	-0.10*	0.05	0.06	-0.03	-0.07	0.16**	0.24****	0.12*	0.72****					
12. Mastery	3.90	1.40	1-7	0.09	-0.14**	0.04	0.02	-0.03	-0.03	0.06	0.25****	0.01	0.53****	0.53****				
13. Control over leisure time	4.93	1.42	1-7	0.00	-0.08	0.09	0.09	-0.10	-0.05	-0.02	0.30****	0.14**	0.53****	0.61****	0.59****			
14. Need satisfaction	5.30	.85	1-7	-0.14**	0.08	0.11*	0.03	-0.03	0.00	0.02	0.16**	-0.01	0.25****	0.31****	0.29****	0.38****		
15. Subjective vitality	4.24	1.27	1-7	-0.12*	0.06	0.07	0.07	-0.03	0.04	0.00	0.23****	0.03	0.23****	0.28****	0.36****	0.35****	0.52****	
16. Perceived stress	3.17	1.17	1-7	0.25****	-0.14**	-0.02	-0.05	0.04	0.02	-0.09	-0.02	-0.06	-0.03	-0.17**	-0.13**	-0.23****	-0.57****	-0.45****

Note. MVPA = Minutes of *Moderate to Vigorous Physical Activity* per week. PA = Physical Activity. *p < .05. **p < .01. ***p < .001. For sex, women were coded 0. For presence to an outside space, no was coded 0. For face-to-face work, “No” was coded 0. For Teleworking, “No” was coded 0.
